# Effects of a pharmaceutical care intervention on clinical outcomes and patient adherence in coronary heart disease: the MIMeRiC randomized controlled trial

**DOI:** 10.1186/s12872-021-02178-0

**Published:** 2021-08-01

**Authors:** Malin Johansson Östbring, Tommy Eriksson, Göran Petersson, Lina Hellström

**Affiliations:** 1 eHealth Institute, Department of Medicine and Optometry, Linnaeus University, Kalmar, Sweden; 2 Pharmaceutical Department, Region Kalmar County, Building 2, floor 2, County Council Hospital, 391 85 Kalmar, Sweden; 3 Department of Biomedical Science, and Biofilm – Research Center for Biointerfaces, Malmö University, Malmö, Sweden

**Keywords:** Pharmaceutical care, Medication adherence, Medicine management, Medication review, Motivational interviewing, Coronary heart disease, Secondary prevention, Low-density lipoprotein cholesterol, Randomized controlled trial

## Abstract

**Background:**

In the treatment of coronary heart disease, secondary prevention goals are still often unmet and poor adherence to prescribed drugs has been suggested as one of the reasons. We aimed to investigate whether pharmaceutical care by a pharmacist at the cardiology clinic trained in motivational interviewing improves clinical outcomes and patient adherence.

**Methods:**

This was a prospective, randomized, controlled, outcomes-blinded trial designed to compare pharmaceutical care follow-up with standard care. After standard follow-up at the cardiology clinic, patients in the intervention group were seen by a clinical pharmacist two to five times as required over seven months. Pharmacists were trained to use motivational interviewing in the consultations and they tailored their support to each patient’s clinical needs and beliefs about medicines. The primary study end-point was the proportion of patients who reached the treatment goal for low-density lipoprotein cholesterol by 12 months after discharge. The key secondary outcome was patient adherence to lipid-lowering therapy at 15 months after discharge, and other secondary outcomes were the effects on patient adherence to other preventive drugs, systolic blood pressure, disease-specific quality of life, and healthcare use.

**Results:**

316 patients were included. The proportion of patients who reached the target for low-density lipoprotein cholesterol were 37.0% in the intervention group and 44.2% in the control group (*P* = .263). More intervention than control patients were adherent to cholesterol-lowering drugs (88 vs 77%; *P* = .033) and aspirin (97 vs 91%; *P* = .036) but not to beta-blocking agents or renin–angiotensin–aldosterone system inhibitors.

**Conclusions:**

Our intervention had no positive effects on risk factors for CHD, but it increased patient adherence. Further investigation of the intervention process is needed to explore the difference in results between patient adherence and medication effects. Longer follow-up of healthcare use and mortality will determine if the increased adherence per se eventually will have a meaningful effect on patient health.

*Trial registration*: ClinicalTrials.gov NCT02102503, 03/04/2014 retrospectively registered.

**Supplementary Information:**

The online version contains supplementary material available at 10.1186/s12872-021-02178-0.

## Background

Coronary heart disease (CHD) is the leading cause of death worldwide [1]. However, mortality and morbidity due to CHD have been more than halved the last decades because of new treatments and methods in the acute phase [2, 3]. Thus, more patients are now treated with secondary prevention drugs such as antiplatelets, cholesterol-lowering drugs and antihypertensives. Despite established guidelines and widespread access to effective medicines, many coronary patients do not reach the treatment goals for blood pressure and cholesterol [4–6]. To be effective, drugs need to be both appropriate for the patients and actually used by the patients, but 20–30% of CHD patients stop taking their preventive medicines at some point after the initiation of treatment [7, 8]. Suboptimal prescribing and poor patient adherence are both associated with increased morbidity and mortality in CHD [8–10]. Twenty-five percent of non-adherent patients have multiple barriers to adherence, the most common being forgetfulness and health beliefs [11]. Patients’ beliefs about their medicines are important determinants of both intentional and unintentional adherence [12–17]. In patients with CHD, concern beliefs related to medicines have been found to increase during the time after the event [18], which could explain the decrease in adherence in these patients [7, 8]. Patients are commonly grateful that medicines provide relief of symptoms and extend life, but are also afraid of adverse effects; thus uncertain of the total net benefit [19, 20]; and coping with medicines can be burdensome and can affect quality of life [21].

The concept of *pharmaceutical care* is based on the responsibility of the caregiver to meet all of the patient’s drug-related needs for the purpose of achieving definite outcomes that improve the patient’s quality of life. When practicing pharmaceutical care, healthcare professionals respect the patient’s personal approach to the use of medicines based on his/her experience; the professionals form a therapeutic relationship with the patient, take responsibility for all the patient’s pharmacotherapy, regardless of source, and focus on the patient’s drug-related needs. Pharmacist interventions (including patient education, feedback to the physician, and medication reviews) can improve risk factor management in patients with cardiovascular disease [22–26]. The reasons for non-adherence are multiple and individual and, therefore, any attempted intervention must have a broad approach to inventorying problems and must allow for individualized problem-solving in order to be effective in a wide group of patients [27]. Motivational interviewing has been used with some effect in medication adherence interventions [28–33] and also specifically when administered by nurses in cardiac care [34]. Skills in motivational interviewing such as affirmations, open-ended questions, and reflections are appropriate for elucidating the status of a patient’s medication use, assessing their beliefs about medicines, and finding their individual resources; all of these aspects are needed to influence the complex behavior of medication adherence and can be used within pharmaceutical care practice.

The Motivational Interviewing and Medication Review in Coronary heart disease (MIMeRiC) trial investigated whether individualized follow-up with motivational interviewing and medication review by a pharmacist at the cardiology clinic improves clinical outcomes and patient adherence [35]. The theoretical framework and the development and evaluation of this pharmaceutical care intervention have been described elsewhere [36].

## Methods

### Trial design and setting

The MIMeRiC trial was a randomized, controlled, outcomes-blinded trial with two parallel groups. Patients were randomized to standard care (control) or standard care plus a follow-up program that included medication review and motivational interviewing (intervention). The primary objective of the trial was to evaluate the achievement of low-density lipoprotein cholesterol (LDL-C) treatment targets by 12 months after discharge in patients receiving the intervention compared with those receiving standard care. The secondary objectives were to evaluate the effects of the intervention on adherence to secondary prevention drugs, systolic blood pressure, health-related quality of life, and secondary healthcare use.

Patients with CHD (International Statistical Classification of Diseases and Related Health Problems, 10th revision [ICD-10] I20-I21) were recruited from the cardiology unit at the County Hospital in Kalmar, Sweden. After inclusion during October 2013 to May 2014 and November 2014 to December 2016, the patients were followed for 15 months. The participants were randomized in blocks of 10, stratified according to their attitudes to their heart medicines, as measured by the Beliefs about Medicines Questionnaire-Specific (BMQ-S) [12, 13] after their standard care follow-up with the physician. Blinding to allocation was not possible due to the nature of the intervention (Additional file 1) [35].

Ethical approval was obtained from the Regional Ethics Committee, Linköping, Sweden, (Dnr-2013/236-31) and all participants gave informed consent. The trial was registered in ClinicalTrials.gov, identifier: NCT02102503, retrospectively registered 03/04/2014.

### Study population

Patients with angiographically verified CHD who were scheduled for follow-up at the out-patient clinic were eligible to participate if they spoke Swedish. Patients were excluded if they had cognitive impairment or any other condition making interviews or phone calls difficult, if they did not participate in the standard follow-up, or if they had prior participation in this study.

### Standard care

Participants in the control group received standard care only. Standard care at the cardiology unit of the County Hospital in Kalmar comprises a 60-min appointment with a cardiac specialist nurse two weeks after discharge, and a 60-min appointment with a resident or cardiologist about two months after discharge. These appointments cover follow-up of hospitalization, information and education about risk factors and medicines, and patient understanding, health status and treatment effects at two months. Unless the patient requires specialist follow-up for other cardiac conditions, e.g. cardiac arrest, pacemaker, or severe heart failure, referral is made to their primary-care facility for continuing follow-up. All patients are also offered cardiac rehabilitation such as physical training in a group at the hospital or at a primary-care facility closer to home.

### Intervention

The participants randomized to the intervention group received a follow-up program run by two clinical pharmacists in addition to standard care. Details of the intervention protocol and the associated theoretical framework have been previously described [35, 36]. The mainstay of the intervention consisted of two appointments at the cardiac out-patient clinic with pharmacists trained in motivational interviewing and medication reviews (Additional file 1). The participants were scheduled for a 60-min appointment with the clinical pharmacist, following their standard follow-up appointments at the clinic, around three months after discharge. The intervention ended with a final follow-up appointment at around ten months after discharge. The basic intervention was offered to all intervention patients, and an intensive intervention with more contact between the first and final visits was offered to patients with specific needs, such as side effects or high concern beliefs about their medicines.

The medication reviews were advanced, type 3, according to the Pharmaceutical Care Network Europe classification [37], and were based on national and European guidelines [38, 39] and the data in Textbox 1. Motivational interviewing was used to find the patient’s specific barriers to adherence and to give useful information about health and medicines only if desired by the patient. The pharmacist arranged the meeting in the spirit of motivational interviewing and pharmaceutical care, i.e. the goal was that patients should feel safe and secure with their medication, and any problems affecting adherence or quality of life would be found and solved together. Thus, an agenda was set to focus the interview on relevant themes (Table 1); any changes were discussed with the patient to assess readiness for change, and the patient was given a written summary of agreed next steps. The pharmacist made a follow-up phone call two weeks after the visit to enquire about the agreed changes, to see if there were new questions, and to strengthen the message from the interview.

**Table 1:** Data collected and reviewed in medication reviews with motivational interviewing

Documentation in the EHR	Baseline questionnaires	Patient interview
Specific diagnosis and treatment decisions for CHD	BMQ-S	Every-day use of medicines Understanding the purpose of medications
Individual risk factors		Thoughts on risks and benefits of medications
Prescribed drugs		Side effects
Medication history		Worries about medicines
Laboratory findings		Earlier medication experience

BMQ-S, Beliefs about Medicines Questionnaire-Specific; CHD, coronary heart disease; EHR, electronic healthcare record

Any drug-related problems that could not be solved by the pharmacist and patient together, such as need of more intensified treatment, were discussed with the cardiologist after the visit, and patients were contacted by phone if prescription changes were made. The intervention protocol was adjusted according to the patient's beliefs about medicines or need for support, i.e. the basic or intensive interventions. If the patient had negative beliefs according to BMQ-S, i.e. ambivalent, skeptical or indifferent, the pharmacist arranged a more thorough interview and offered the patient more visits or continued contact by phone. However, patients with an accepting attitude were also offered extra contact opportunities if their concerns were revealed in the interview, or if they had side effects or ineffective treatment. The more intensive intervention protocol offered the patient up to four extra contacts, either in person or by phone, as an extension of the first visit; patients with side effects or concerns about their medicines were followed until these problems were resolved or the patient and the pharmacist agreed that no more follow-up was necessary. All patients were scheduled for a final follow-up visit of approximately 20–30 min. This aimed to support the patients for their subsequent “lifelong” medicine use and to guide them to obtain follow-up support at a primary-care facility, if they had no established primary-care contact already. Before the appointment, the pharmacist reviewed any changes in health status and prescribing in the electronic healthcare record (EHR), monitored the lipid profile (the patient received a referral for a laboratory test along with the scheduled appointment), and re-assessed the patient's beliefs about their medicines. A written summary and a follow-up phone call were made only if new problems were encountered. Any problems found at this stage were communicated to the primary-care physician, either through referral or with a personal message in the EHR.

### Outcomes and data collection

Lipid status and blood pressure were assessed 12–14 months after discharge as outlined in the Swedish national quality register for secondary prevention (SEPHIA). Data on LDL-C, systolic blood pressure, prescription refills, and number of healthcare contacts were obtained as a report from the EHR database*.* At 15 months post-discharge, participants were asked to complete the same questionnaires as those they completed at baseline; see study protocol for details [35].

#### Primary outcome

The primary outcome was the proportion of patients who reached the treatment goal for LDL-C levels. The treatment goal, as assessed by SEPHIA, was an LDL-C of < 1.8 mmol/L (corresponding to 70 mg/dL), or a reduction of 50% from the level prior to statin treatment [40]. LDL-C values were calculated from the serum concentrations of cholesterol and fasting triglycerides, using the Friedwald formula.

#### Secondary outcomes

Patient adherence to cholesterol-lowering drug regimens was the key secondary outcome. The proportion of patients who adhered to the treatment regimen was assessed using two methods. These focused on the implementation and persistence phases of treatment, as defined by the “ABC-taxonomy for medication adherence” [41]. Because self-reporting and refill adherence methods each have associated disadvantages, they were combined [42]. Thus, the patient was considered non-adherent if either method suggested non-adherence. See Additional file 1 for an overview of adherence measures and outcome definitions.

Self-reported adherence to cholesterol-lowering drug regimens was assessed with the Morisky 8-item adherence scale (MMAS-8, (license obtained)) [43–45], for which license was obtained. For refill adherence, patients were defined as non-persistent at 15 months post-discharge if they had not purchased the drug at least once during the 12- to 16-month period after discharge, as long as they had a valid prescription during this period. We obtained both refill data, which are continuously transferred from the Swedish Prescribed Drug Register to the EHR database, and prescription data from the EHR database. The proportions of patients who were persistent in refilling prescriptions for cholesterol-lowering drugs, aspirin, beta-blocking agents, angiotensin-converting enzyme inhibitors, and angiotensin receptor blockers (RAAS inhibitors) were assessed this way. A third adherence estimate, implementation adherence, was also used for cholesterol-lowering drugs: the proportion of days covered (PDC). A cut-off point of 80% was set for the PDC [46].

To further investigate the relationship between adherence and the primary outcome, we tabulated the LDL-C outcome with the dichotomous PDC outcome, which is the adherence measure relevant for the time period precluding and overlapping the time for LDL-C follow-up. Beliefs about medicines were assessed using the BMQ-S and were used as a process measure; however, to aid interpretation of the results of this intervention we have also reported the summary scores and attitude categories of the BMQ-S. Detailed analysis of beliefs will be reported together with other process measures in a separate manuscript [36].

Secondary outcomes also included the following: the proportion of patients with systolic blood pressure < 140 mmHg 12 months after discharge; participants’ quality of life assessed with the disease specific health-related quality of life questionnaire (HeartQoL) [47]; and the number of patients with emergency visits or hospitalizations due to cardiovascular disease (ICD I00-99 and Z034).

### Sample size

The initial sample size calculation indicated that we would need 195 participants in each group to detect a shift in the proportion achieving the primary goal for LDL-C from 0.3 to 0.5 and a difference of 10% in the proportion of patients with refill adherence, with 80% power at a significance level of *P* = 0.05 (two-sided). Changed circumstances resulted in a new sample size calculation in 2016, see published protocol for details [35]. The new calculation was based on a shift in the proportion reaching the primary goal from 0.45 in the control group to our expectation of 0.6 in the intervention group. This meant that 170 patients were needed in each group for 80% power to reject the null hypothesis, or 134 patients for 70% power, for the primary outcome. No new calculations were made for the adherence outcome, but 170 patients would correspond to at least 70% power for this outcome.

### Statistical analyses

All the data were analyzed using IBM SPSS 24.0 software. All tests were two-sided and exact *P*-values were calculated. The analyses of participants and non-participants and of baseline characteristics were conducted using independent sample *t* test and chi-square tests. In these analyses *P*-values were calculated for descriptive and not inferential purposes. As stated in the protocol, intervention effects should be tested using logistic or linear regression analyses with baseline values of the outcome variable as covariates. However, since no baseline values were present for adherence measures, we decided to calculate the risk difference instead. For categorical variables with baseline values, LDL-C, blood pressure and proportion of patients with healthcare contacts, sensitivity analyses using logistic regression with adjustment did not change the conclusion. We have therefore reported the risk difference for all the categorical variables and the *z*-test was used to compare groups. Three outcome variables that were not described in the study protocol, LDL-C mean values at follow up, refill adherence for cholesterol-lowering drugs related to total study group, and PDC mean values, are reported as descriptive variables to guide interpretation. An independent sample *t* test was used for LDL-C in cross-tabulation of the adherence measures, and chi-square tests were used for categorical variables unless the expected numbers were small, when Fishers exact test was used.

At the time of analysis, it was apparent that there were considerable amounts of missing outcome data, not as a result of participants dropping-out completely, but because some variables were missing for many patients. Complete outcome data were available for only 48.5% of patients, and it was therefore decided to analyze each variable separately. The amounts of missing data varied among the variables, and tended to be higher for the intervention group for questionnaire data (22.0% vs 13.4% for HeartQoL; *P* = 0.044). Multiple imputations or linear mixed models were not used to handle missing data because of considerable amounts of missing data for several variables, and because there were no auxiliary variables. Because of this, multiple imputations would have had a marginal effect, would have been of limited value for interpreting the findings, and would have added unnecessary complexity [48–50]. We found that the patients with missing LDL-C values were slightly older and a larger proportion were living alone; participants failing to provide a blood pressure measurement were older too.

We assumed that the missing values were mostly missing at random (MAR), but it was not implausible that they were missing not at random (MNAR). We conducted an intention-to-treat (ITT) analysis based on all collected data but with complete cases per variable, meaning that for each variable we used data from all patients who completed this measure (that is, the assumption was that the data were missing completely at random; MCAR). We tried to make this complete case per variable analysis more compatible with the MAR assumption by adjusting for age and living alone [50]. Since the result of the primary outcome was unaffected by the adjustment, we have reported the unadjusted results only. To fulfill the ITT analysis strategy proposed by White et al. [51], we then performed sensitivity analyses with different clinically plausible departures from the MAR assumption, considering all randomized participants. We also performed sensitivity analyses to determine how robust the results were to different assumptions about the outcomes definition. For details about missing data and sensitivity analyses see Additional files 1 and 3 respectively. Missing individual items in questionnaires were imputed with a simple mean imputation, if the numbers did not exceed 20% [52]. In total, values were replaced in 11, 26, and 39 observations in the BMQ-S, MMAS-8 and HeartQoL questionnaires, respectively.

## Results

**Fig. 1:**
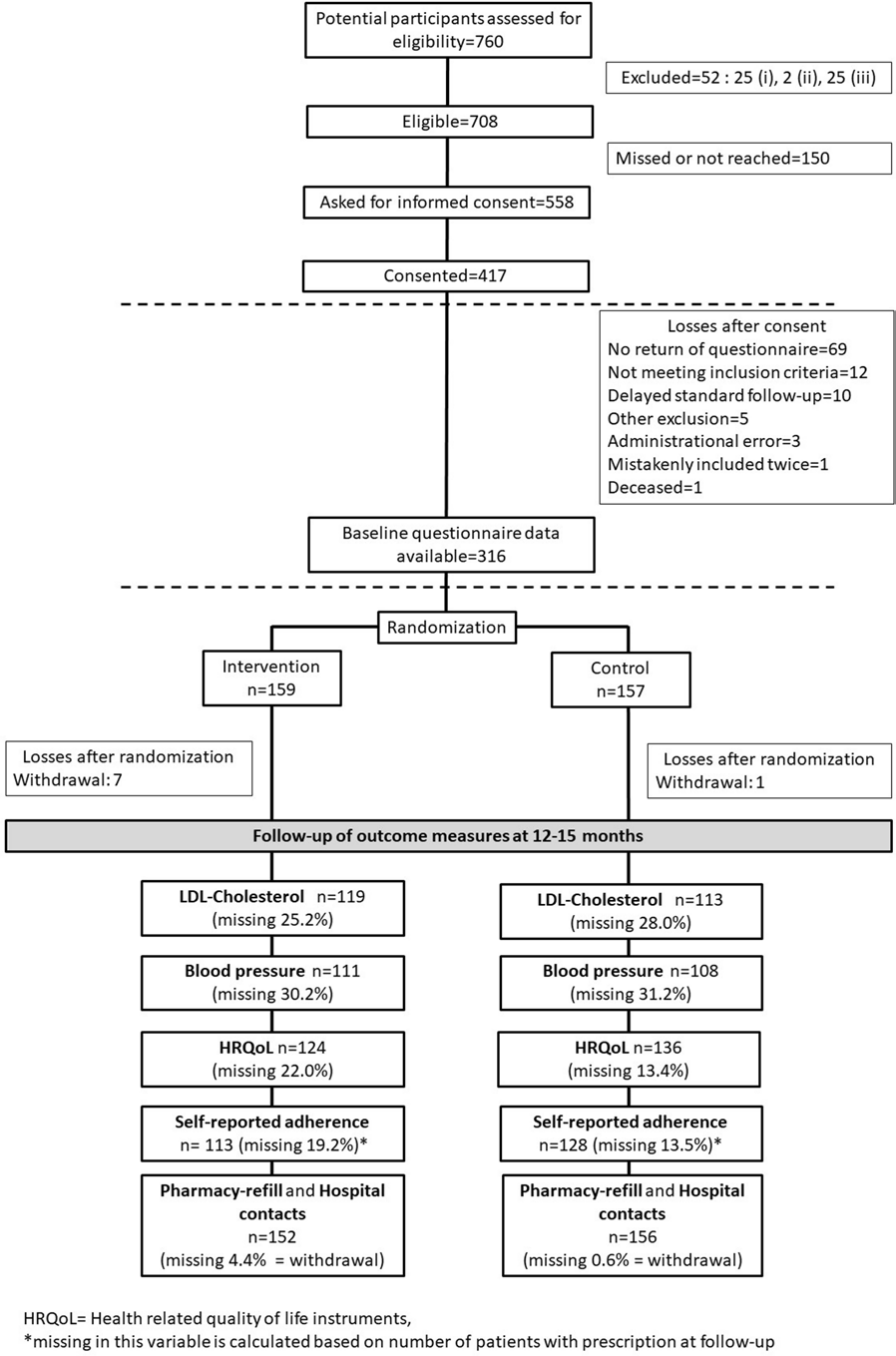
Study flowchart. For those excluded, (i) indicates cognitive impairment or any other condition making interviews or phone calls difficult; (ii) indicates nonparticipation in the standard follow-up at the out-patient clinic; and (iii) indicates prior participation in this study

Figure 1 provides an overview of the flow of participants in the study. In total, 708 patients were eligible for the study of which 316 were included: 157 in the control group and 159 in the intervention group. For details about participants and non-participants, see Additional file 2. At baseline, patient and clinical characteristics were similar in the control and intervention groups, see Table 2. Prescribed medicines for cardiovascular disease at discharge is reported in Additional file 2. All patients randomized to intervention (n = 159) were summoned for a first visit, and 144 completed this, a flowchart of intervention activities is available in Additional file 2.

**Table 2:** Baseline characteristics of participants

	Intervention group (n = 159)	Control group (n = 157)	*P*
Age (years), mean ± SD	68.3 (8.9)	68.6 (8.6)	.744
Male, n (%)	116 (73.0)	119 (75.8)	.563
Born outside Sweden, n (%)	10 (6.3)	11 (7.0)	.798
Married or cohabitating, n (%)	126 (79.2)	122 (78.2)	.737
Educational level, n (%)			.750
Comprehensive school	56 (35.2)	60 (38.2)	
Upper secondary school	43 (27.0)	37 (23.6)	
Bachelor’s/Master’s degree	60 (37.7)	60 (38.2)	
Type of CHD, n (%)			.834
STEMI	46 (28.9)	48 (30.6)	
Non-STEMI	48 (30.2)	43 (27.4)	
Unstable angina	17 (10.7)	18 (11.5)	
Chronic angina	32 (20.1)	37 (23.6)	
Other reason for PCI	15 (9.4)	10 (6.4)	
History of CHD, n (%)	47 (29.6)	45 (28.7)	.919
Unplanned healthcare contact for CVD, preceding year, n (%)	27 (17.0)	20 (12.7)	.289
Coronary intervention, n (%)			.459
PCI with DES	126 (79.7)	110 (72.8)	
Other PCI	12 (7.6)	12 (7.9)	
CABG	10 (6.3)	15 (9.9)	
None	10 (6.3)	14 (9.3)	
Comorbidities, n (%)			
Hypertension	79 (48.4)	74 (49.0)	.910
Other CVD	22 (13.8)	17 (10.8)	.392
Diabetes	23 (14.5)	20 (12.7)	.622
Other comorbidities	60 (37.7)	70 (44.6)	.303
No comorbidities	35 (22.0)	33 (21.0)	.830
Clinical risk factors, n (%)			
Smoking	11 (8.0)	17 (12.6)	.080
Smoking eligible participants, n	137	135	
Mean LDL-C, mmol/L (SD)	2.2 (0.8)	2.3 (0.8)	.909
LDL -C, treatment target reached, n (%)	55 (39.6)	58 (41.7)	.714
LDL-C eligible participants, n	139	139	
Mean SBP mm Hg (SD)	139.5 (20.6)	138.1 (19.6)	.526
SBP, treatment target reached, n (%)	79 (52.3)	90 (58.1)	.312
Mean DBP, mm Hg (SD)	76.1 (11.6)	75.1 (11.7)	.460
BP eligible participants, n	151	155	
Number of drugs prescribed per participant			
For regular use, mean (SD)	7.2 (2.4)	7.3 (2.3)	.871
As needed, mean (SD)	2.1 (1.2)	2.1 (1.3)	.899
Self-reported adherence^a^			
MMAS-8 score (SD)	7.5 (1.2)	7.5 (1.0)	.889
MMAS-8 medium or high adherence, n (%)	132 (93.0)	137 (91.9)	.744
MMAS-8 eligible participants, n*	142	149	
Beliefs about medicines			
Mean Necessity score (SD)	19.0 (3.1)	19.0 (3.1)	.913
Mean Concern score (SD)	13.0 (4.9)	13.1 (5.1)	.884
Mean Necessity-Concerns differential (SD)	6.0 (5.7)	5.9 (6.3)	.964
BMQ-S attitudinal category			.359
Accepting, n (%)	79 (51.0)	82 (53.2)	
Ambivalent, n (%)	54 (34.8)	50 (32.5)	
Indifferent, n (%)	11 (7.1)	7 (4.5)	
Skeptical, n (%)	8 (5.2)	15 (9.5)	
HeartQol score			
Mean HeartQoL physical (SD)	2.2 (0.8)	2.2 (0.7)	.926
Mean HeartQoL emotional (SD)	2.4 (0.7)	2.3 (0.8)	.178
Mean number of days from discharge to randomization (SD)	116.4 (37.7)	122.5 (51.0)	.226

BP, blood pressure; BMQ-S, Beliefs about medicines questionnaire Specific; CABG, Coronary artery by-pass grafting; CHD, coronary heart disease; CVD, cardiovascular disease; DES, drug-eluting stent; DBP, diastolic blood pressure; HeartQoL, health-related quality of life; LDL-C, low-density lipoprotein cholesterol; MMAS-8, Morisky 8-item adherence scale; n, number; PCI, percutaneous coronary intervention; SBP, systolic blood pressure; STEMI, ST-elevation myocardial infarction

*This questionnaire was only to be filled in by those with a prescription for a cholesterol-lowering drug

^a^The use of MMAS diagnostic adherence assessment instrument is protected by US copyrighted and trademarked laws. Permission for use is required. A license is available from—MORISKY MEDICATION ADHERENCE RESEARCH, LLC., Donald E. Morisky, ScD, ScM, MSPH, MMAR, LLC, 294 Lindura Ct., Las Vegas, NV 89138; dmorisky@gmail.com

### Primary outcome

The proportion of patients who reached the target for LDL-C was 37.0% in the intervention group and 44.2% in the control group; absolute difference -7.2% (95% CI -19.9% to 5.3%). The mean LDL-C concentration at follow-up was 2.3 vs 2.2 mmol/L in the intervention and control groups, respectively (Table 3). Around 25% of patients did not have a follow-up laboratory evaluation of LDL-C.

**Table 3:** Primary outcome

LDL-Cholesterol	Intervention group (n = 159)	Control group (n = 157)	Risk difference, % (95% CI)	*P*
LDL-C < 1.8 mmol/L,* n (%)	44 (37.0)	50 (44.2)	-7.2 (-19.9 to 5.3)	.263
LDL-C (mmol/L), mean (SD)	2.3 (0.7)	2.2 (0.7)		
eligible participants, n (%)	119 (74.8)	113 (72.0)		

*Or 50% reduction from pre-treatment value, if this could be identified

LDL-C, low-density lipoprotein cholesterol; n, number; SD, standard deviation

### Secondary outcomes

#### Patient adherence

**Fig. 2:**
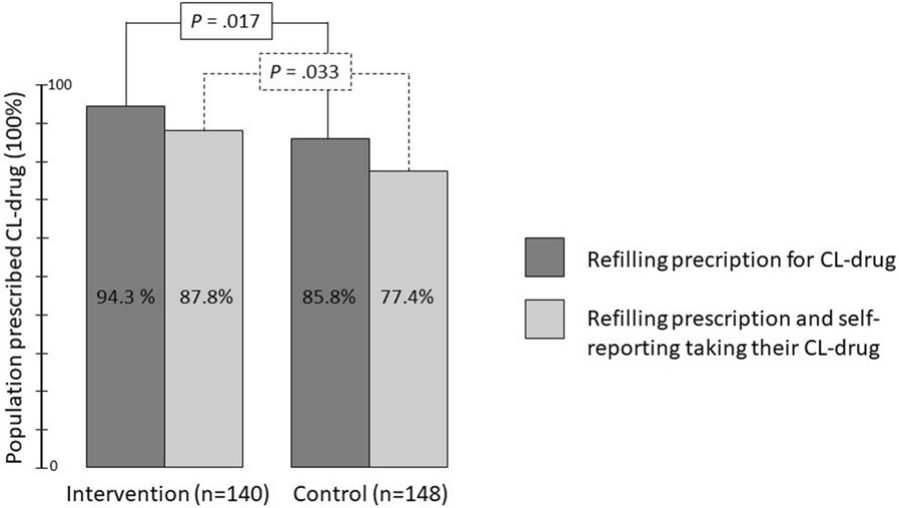
The proportion of patients who refilled their prescription for cholesterol-lowering (CL) drugs, and the proportion of patients who both refilled their prescription and reported adherence to their drug regimen

A similar proportion of patients in the intervention and control groups were prescribed cholesterol-lowering drugs (92.1 vs 94.9%) at follow-up, and the proportions prescribed a high-intensity statin were 67.1% and 67.9%, respectively. More patients were adherent to their regimens of cholesterol-lowering drugs in the intervention group than in the control group (87.8% vs 77.4%) according to the combined measure of adherence, i.e. there was an absolute risk difference of 10.4% (95% CI 1.1% to 19.7%). A summary of all adherence results is provided in Table 4. When adherence was measured as an individual variable, more intervention patients than control patients were persistent according to refill adherence (absolute risk difference 8.5%; 95% CI 1.7% to 15.3%), but the association between group and self-reported implementation adherence was weaker. Implementation adherence during the intervention and follow-up period, measured as PDC, was similar in the intervention and control groups (PDC ≥ 80%, 83.6% vs 79.5%). The proportion of patients who refilled their prescriptions for cholesterol-lowering drugs, and the proportion who both refilled their prescription and reported adherence (MMAS-8) to their prescribed drug regimen are shown in Fig. 2. More patients in the intervention group (97.1%) than in the control group (91.2%) were persistent to their aspirin regimens, i.e. the absolute risk difference was 5.9% (95% CI 0.5% to 11.3%). We found minor differences between the groups in persistence to beta-blocking agents or RAAS inhibitors.

**Table 4:** Secondary outcomes, adherence and beliefs about medicines

Adherence measures	Intervention (n = 152*)	Control (n = 156*)	Risk difference, % (95% CI)	*P*
*Adherence measures for CL drugs*				
Participants prescribed CL drug, n (%)	140 (92.1)	148 (94.9)		
Participants with a positive combined adherence measure, n (%)	101 (87.8)	103 (77.4)	10.4 (1.1 to 19.7)	.033
Eligible participants, n	115	133		
MMAS-8 score (SD)	7.6 (0.8)	7.4 (1.4)		.117
MMAS-8 medium or high adherence, n (%)	106 (93.8)	115 (89.8)	3.9 (-2.9 to 10.9)	.267
Eligible participants, n	113	128		
Participants who refilled a prescription for CL drug, n	132	127		
As a proportion of those with prescription, %	94.3	85.8	8.5 (1.7 to 15.3)	.017
As a proportion of total study group, %	86.8	81.4	5.4 (-2.7 to 13.6)	
Participants with PDC 0–15 months ≥ 80%, n (%)	116 (83.6)	112 (79.5)	1.3 (0.8 to 2.4)	.376
PDC 0–15 months, mean (SD)	89.6 (12.6)	89.1 (13.8)		
Eligible participants, n	134	146		
*Refill adherence measure for ASA, BB, RAASi*				
Participants who refilled a prescription for ASA, n	133	134		
As a proportion of those with prescription, %	97.1	91.2	5.9 (0.5 to 11.3)	.036
Participants who refilled a prescription for BB, n	120	121		
As a proportion of those with prescription, %	91.6	90.3	1.3 (-5.6 to 8.2)	.711
Participants who refilled a prescription for RAASi, n	123	129		
As a proportion of those with prescription, %	93.9	92.1	1.7 (-4.3 to 7.8)	.575
*Beliefs about medicines*				
Eligible participants	126	137		
Mean Necessity score (SD)	19.1 (3.1)	18.7 (3.5)		.324
Mean Concern score (SD)	11.2 (4.6)	12.5 (4.8)		.035
Mean Necessity-Concern differential (SD)	7.9 (5.7)	6.3 (5.8)		.022
BMQ-S attitudinal category				.340
Accepting, n (%)	79 (62.7)	81 (59.1)		
Ambivalent, n (%)	34 (27.0)	35 (25.5)		
Indifferent, n (%)	11 (8.7)	13 (9.5)		
Skeptical, n (%)	2 (1.6)	8 (5.8)		

ASA, acetylsalicylic acid; BB, betablocking agent; BMQ-S, Beliefs about medicines questionnaire Specific; CL, cholesterol-lowering; ITT = intention-to-treat; n, number; PDC, proportion of days covered; RAASi, renin–angiotensin–aldosterone system inhibitors

*Number of participants in intervention and control groups 95.6% and 99.4% of ITT groups (ITT n = 159 and 157)

Beliefs about medicines are also reported in Table 4. Patients in the intervention group had lower concern scores than patients in the control group (11.2 (4.6) vs 12.5 (4.8); *P* = 0.035), and a more positive necessity-concerns differential. There was no difference between groups in the proportion of patients in the different attitudinal categories.

Patients who reported poor implementation adherence (MMAS-8) to cholesterol-lowering regimens were less likely to be persistent (to have refilled their prescription), but the majority (n = 14, 63.6%) of patients who were not persistent (did not refill their prescriptions) reported good implementation according to the MMAS-8. Few (n = 15, 6.8%) of the patients who refilled their prescriptions reported poor implementation. In the intervention group, patients with PDC ≥ 80% had lower LDL-C levels than patients with PDC < 80%; the mean LDL-C concentration was 2.1 (0.7) mmol/L vs 2.5 (0.7) mmol/L, respectively (*P* = 0.049). There was no difference in the control group. However, in both groups there was a trend that a larger proportion of the adherent patients than the non-adherent patients reached the LDL-C goal; see Additional file 2 for details.

#### Clinical outcomes

A summary of the secondary clinical outcomes results is provided in Table 5. A similar proportion of patients in each group reached the treatment target for systolic blood pressure. Patients in both groups reported that their emotional and physical quality of life was the same as before the intervention. 14.5% of patients in the intervention group vs 9.0% in the control group (absolute risk difference 5.4%; 95% CI -1.7% to 12.6%) had unplanned contact with healthcare for cardiovascular disease (CVD) during this follow-up period.

**Table 5:** Secondary outcomes, clinical outcomes

Categorical variables	Intervention group (n = 159)	Control group (n = 157)	Risk difference, % (95% CI)	*P*
Systolic blood pressure < 140 mm Hg, n (%)	66 (59.5)	63 (58.3)	1.1 (-11–9 to 14.2)	.865
Eligible participants, n	111	108		
Unplanned healthcare contact, n (%)	22 (14.5)	14 (9.0)	5.4 (-1.7 to 12.6)	.138
Eligible participants, n	152	155		

Continuous variables, mean (SD)			B (95% CI)	*P*
HeartQoL physical score	2.3 (0.7)	2.3 (0.7)	0.044 (-0.080 to 0.169)	.485
HeartQoL emotional score	2.5 (0.6)	2.4 (0.7)	-0.002 (-0.126 to 0.121)	.970
Eligible participants, n	124	136		

B, unstandardized coefficient; HeartQoL, Heart quality of life instrument; n, number; SD, standard deviation

#### Per-protocol and sensitivity analyses

There were no evident changes in the primary outcome between the ITT and the per protocol analysis. The results of the adherence tests were reinforced for the intervention group when only patients who had received the full intervention were included, i.e. per protocol (n = 130), see Additional file 2 for details.

Sensitivity analyses of the primary outcome showed that under a MAR condition there would be no change of conclusion. If the values were missing not at random, the conclusion of the trial would be altered to a negative result for the intervention group if the imputed values were < 30% goal achievement in the intervention group and > 50% in the control group. Sensitivity analyses were also carried out for the two adherence measures with significant risk differences, and the results were found to be robust to different assumptions about missing data. All sensitivity analyses are reported in Additional file 3.

## Discussion

The study suggests that a pharmaceutical care intervention with medication reviews and motivational interviewing by a clinical pharmacist as part of secondary prevention care in patients with CHD improved medication adherence, possibly through an effect on medication beliefs. However, the intervention did not improve the clinical outcomes for LDL-C or blood pressure, nor for quality of life or secondary healthcare use in the first 15 months after discharge.

We found that the intervention lowered patients’ concerns about medicines and that patients in the intervention group were more adherent to cholesterol-lowering and aspirin regimens. The combined result of improved medication beliefs and improved adherence behavior has been found in a few other trials [53–56]. These trials have also tested complex interventions tailored to patients’ individual adherence barriers, but in other patient groups and with other adherence measures. Given the evident impact of beliefs on adherence behavior [13, 57], our study adds to this ‘proof-of principle’ that person-centered consultations on medication use, specifically targeting medication beliefs, can effectively change adherence behavior.

In our study, the persistence rate after one year in the control group was around 80% for cholesterol-lowering drugs, and this is similar to adherence rates found in recently published Swedish cohorts [58, 59]. Given our assumptions, the results suggested a 10% increase in adherence to cholesterol-lowering drugs in the intervention group, but a difference ranging from 1 to 20% was also compatible with our data. This is in line with other multifaceted interventions in similar patients which have often shown a risk difference of about 10% [33, 60, 61] and occasionally more [62, 63] in their respective refill adherence measure. In our study, although the intervention patients were more adherent to cholesterol-lowering drugs, they did not reach the treatment target more often than control patients. In fact, the results for the primary outcome (LDL-C) indicated a trend for lower goal achievement in the intervention group than in the control group, even though mean LDL-C levels were similar.

This contradictory finding of better adherence but lower goal attainment for LDL-C could have several explanations. Firstly, it is important to consider that the LDL-C concentrations were measured before the adherence assessment at the 15-month follow-up, in which we found adherence improvements for the intervention group; in contrast, there were no improvements in implementation adherence (PDC 0–15 months). Thus, the measurement of LDL-C in our study may not have been preceded by increased adherence. Secondly, the effect on adherence but lack of corresponding effect on the clinical outcome has been found in several earlier adherence interventions [33, 60, 62]; this might be because the adherence and outcome measurements were followed-up at the same time, while the effect of increased adherence may only become apparent in the longer perspective. As pointed out by Ho et al. [62], this was demonstrated by Choudhry [63], one of the largest studies to measure adherence and major vascular events. The proportion of patients in that study who had PDC ≥ 80% was about 10% higher among patients with full insurance coverage for their medicines, but the curves for the outcome started to diverge after one year of different adherence rates. Therefore, it could be misleading to state that interventions that increase adherence to cardiovascular drugs have no potential for improving patients’ long term health, based on a failure to detect differences in clinical outcomes at the time of adherence follow-up.

Regarding treatment quality, there was no difference between the groups in terms of the proportion prescribed high-intensity statins at follow-up and, therefore, it could appear that the pharmacists were not actively suggesting intensification of the treatment. On the other hand, the overall aim of the intervention was to improve secondary prevention treatment in a framework of pharmaceutical care, i.e. to achieve better patient outcomes and to improve the quality of each patient’s life by optimizing the drug therapy in cooperation with the patient [36]. Thus, the pharmacists might have taken responsibility for a longer period, i.e. supporting the patients and balancing the effects and side effects of drugs in a way that could improve long term adherence/persistence, but that would mean less intensive therapy. Adding to this, there have been different stands on the importance of reaching a specific LDL-C value as opposed to using a specific statin dose [64, 65]. This could have influenced the practice of the pharmacists conducting the intervention, which could in turn have driven the results towards higher adherence but with fewer patients reaching an LDL-C of 1.8 mmol/L. The process evaluation of this trial will provide insight in this matter; we will then assess intervention fidelity and analyze the actions taken by pharmacists in the medication reviews [36].

Although it is unclear how adherence interventions impact on the clinical outcomes of CHD [33, 62, 63], we know that patients with poorer adherence have a greater risk of cardiovascular morbidity and mortality [59] as well as all-cause mortality [9, 58, 59]. Thus, in our study, the increased persistence to statin treatment in the intervention group might in itself have an impact on future morbidity and mortality; based on Swedish cohorts [58, 59], we believe that a 10% increase in persistence is clinically relevant. The higher persistence rate for aspirin in the intervention group should also confer a decreased risk of long term morbidity [66] and mortality [67]. However, it is important to stress that our study’s hypothesized effect on morbidity as a result of increased adherence needs longer follow-up, so that the effect can be related to persistence at 15 months. As described earlier [35], we will analyse this again for the 36-month follow-up results.

The intervention appears to have had an effect on adherence to cholesterol-lowering drugs and aspirin only, which may be explained by the intervention itself. An essential part of the intervention was to inform patients on the purpose of different medicines and to ascertain that patients knew which medicines were most important. For many of the patients who did not have heart failure or major hypertension, the aspirin (along with the antiplatelet agent which they had stopped taking at follow-up) and cholesterol-lowering medications were the most important drugs. However, the difference between therapeutic groups could also be attributed to details about measurement. Compared to aspirin, the anti-hypertensive drugs are more commonly paused for the investigation of suspected side-effects; something that could typically happen during the intervention. Because our measurement did not take account of such pauses, persistence might have been underestimated in the intervention group.

### Comparison with related studies

There are few earlier studies with a similar intervention design which have investigated effects in patients with cardiovascular disease. However, our intervention was in some ways similar to a multifaceted intervention tested by Ho [62] which increased implementation adherence to secondary preventive drugs. As in our study, the adherence effects did not result in higher goal achievement for LDL-C or blood pressure, nor in fewer re-hospitalizations, a failure common among adherence interventions [33, 68]. Are these interventions making more patients adherent, but adherent to a treatment that does not have an impact on risk factors? Our data, in line with Ho [62], do not support this, but rather suggest that patients who are adherent in the intervention group have better risk factor control than the non-adherent patients. Thus, patients who are affected by the intervention have better outcomes. However, the proportion of patients who became adherent because of the intervention was too small for this difference in LDL-C to become apparent and detectable in a comparison based on the complete intervention and control groups.

The use of motivational interviewing to increase adherence and/or risk factor control in CHD patients has been tested in a few recent studies with varied results; some found an impact on adherence but not on risk factor control [69], others did not find evidence of any effect on adherence [70, 71]. However, one study by Lin et al. [61] found that an intervention similar to but more intense than ours had effects on adherence but, in contrast to ours, it also had significant effects on LDL-C, quality of life and survival rates. Thus, in a setting with patients with higher cardiovascular risk, higher risk of non-adherence and higher lipid levels, the intervention improved both adherence and clinical outcomes. We suggest that the differing results among these studies might relate, among other things, to the different professionals conducting the interventions. Our intervention, along with those by Ho et al. [62] and Lin et al. [61], expanded the multidisciplinary team by the addition of another profession, adding skills and experience, such as those of conducting a medication review and practicing pharmaceutical care.

This is in line with the evidence for adherence interventions in general: a meta-analysis of 771 trials found that the most effective interventions were delivered face-to-face by pharmacists [72]. In cardiovascular disease, the roles of different professions in improving patient adherence are yet to be established, but interventions by nurses or pharmacists, initiated in the inpatient setting and including phone contact seem especially effective [73–75]. A nurse-led phone-based intervention in a very similar population and setting aimed at controlling risk-factors: cardiac nurses titrated medicines as needed after an annual follow-up of risk factors while control group patients were followed in primary care [76]. This intervention was found effective for LDL-C at the one-year follow-up [76], and after a mean 3.9 years of follow-up intervention group patients had mean LDL-C 2.0 mmol/L compared to 2.4 mmol/L in the control group. Patients in the intervention group were also more likely to stay on statin treatment, even though they also had a more intensive regimen [77]. The authors conclude that the continual patient-centered follow-up enables a joint trust and understanding of symptoms and causes, which can help prevent discontinuation of statin treatment.

Effective adherence interventions need to target multiple adherence barriers [11, 78, 79] and be flexible enough to support patients based on how the adherence behavior and its underlying reasons change over time [78, 80]. Many studies have attempted to find out whether a basic adherence intervention is sufficient and/or whether a more intensive version would be beneficial [69–71]. These studies have often had problems with uptake in the more intensive arm and, therefore, an intervention design that enables individual intensification and customized content has been suggested as a promising option [69–71, 79]. In our study, we made use of the knowledge that patients’ needs differ with regard to adherence support, that they can change over time, and that the needs or the level of support for each patient cannot be estimated before the first consultation. Thus, the intervention in this study was tailored to patient need, i.e. that patients were given different doses in the intervention. This is, to our knowledge, the first study in this field to test an adherence intervention with individualized intensity. We will report further details about the intervention intensity and different outcome and process measures in the process evaluation paper [36].

### Methodological considerations

The use of a combined adherence measure worked well in this study. If we had relied on refill only, we would have missed 15 patients who had low adherence in MMAS-8, and if we had relied on self-report only, we would have missed 14 patients who reported medium or high adherence even though they did not refill their prescriptions. The draw-back of refill adherence measurement, i.e. that patients might refill but never use their drugs, was to some extent counteracted by the self-report results. However, we still would not be able to find those who refilled their drugs and reported that they used them when they did not. Hence, we believe that the combination of measures is a pragmatic method of approaching real patient adherence.

A broad range of CHD patients was included in this study, i.e. not only those with acute disease but also those who had had previous experience of, and treatment for, CHD. This was because all CHD patients had the same follow-up at our hospital in standard care at the time of the study, all CHD patients were expected to benefit from the intervention, and the required sample size would be easier to attain. However, there might be differences in how the intervention should be delivered and designed for acute and chronic CHD (angina) patients, and for those with a history of CHD. Therefore, the different needs of the diverse study population might have affected the result of our study. We will report the outcomes of different sub-groups in the process evaluation paper [36].

Our study had some limitations. We needed a longer follow-up of LDL-C concentrations to relate the effect on this measure with the intervention’s effect on adherence. Also, the follow-up assessment was only about five months after the end of the intervention, which was relatively short for an adherence intervention on preventive medicines. In future intervention studies regarding mainly preventive drugs, we suggest that adherence is measured for a time period or at a time point precluding the follow-up of morbidity and mortality.

Wide, simple selection criteria were used to make the study results generalizable but unfortunately only half of the eligible patients took part. In particular, the study failed to include younger patients with higher LDL-C levels who lived alone and did not have a university education. Thus, the study failed to include those who would possibly have the greatest need for this kind of intervention. However, the adherence rate in our study is comparable to that in national cohorts and the study results might therefore be generalizable in terms of adherence to other populations, as was seen in a similar trial [81].

Although, we used standardized procedures for the medication review, and motivational interviewing is a well described method, the results may not be fully generalizable to other settings because the study involved only two clinical pharmacists and one particular clinic. An important limitation of our study was the high rate of missing data for the non-registry-based variables, meaning that these results are more explorative than confirmative.

Our intervention was designed to encompass all aspects of adherence, but some barriers to adherence, such as socio-economic factors, are more difficult for healthcare professionals to affect. Future studies might include a tool-kit for this kind of barrier. Depression is a known barrier to adherence, and is quite common in patients with CHD; screening for depression could be included in future interventions in this patient group [82]. The specific needs of patients with a negative attitude towards medicines, and how they should be supported, also warrant further study; this could be related to how different personality traits confer higher risks for non-adherence [83, 84]. In the process evaluation, we also need to explore why some patients were still non-adherent despite the intervention activities.

### Clinical implications

CHD-patients experiences with using medicines differs widely, and they often need better dialogue with healthcare providers to manage their medicines [85]. These patients often experience medication-related burden on different daily life aspects [86], and this relates in part to the number of drugs [87]. Since 2018 new ESC guidelines have been issued on both blood pressure [88], lipid-lowering (the treatment goal is now LDL-C < 1.4 mmol/L) [89], and how to treat CHD-patients with diabetes or prediabetes [90]; all of these imply intensified drug treatment and/or include more patients to be treated with drugs. This increases the need for pharmaceutical care and individualized follow-up assessing both clinical outcomes and the patient’s medication experience [91]. A continual patient-centered follow-up enables a joint trust and understanding of symptoms and causes, which can help prevent discontinuation of treatment [77]. Our study shows that including clinical pharmacists trained in motivational interviewing is one option to deliver support for patients in their management of medicines; and when this is based on patients’ individual needs, they commonly need two-five contacts attributed to therapeutic problems, their attitude towards medicines and their experiences of using them in daily life.

## Conclusions

In patients with CHD who were treated with secondary prevention medications, a medication review and motivational interviewing carried out by a clinical pharmacist during the first year of treatment had no positive effects on the proportion reaching treatment targets, nor on quality of life or healthcare use. However, this intervention based on pharmaceutical care increased adherence to cholesterol-lowering drugs and aspirin by 15 months after discharge and patients’ beliefs about medicines were also positively impacted by the intervention. Further investigation of the intervention process is needed to explore the difference in results between patient adherence and medication effects in this trial, and also to explore pharmaceutical care activities and effects. Longer follow-up of healthcare use and mortality will determine if the increase in adherence has a measurable effect on patient health.

## Supplementary Information

**Additional file 1. Methods** Details about randomization and blinding, intervention pharmacists' education, adherence outcomes, and missing data.

**Additional file 2. Additional results** Characteristics of non-participants, drugs prescribed at discharge, participants and activities in intervention, relation between adherence measures and between adherence and LDL-C, per-protocol analysis.

**Additional file 3.** Sensitivity analyses of primary outcome and relevant secondary outcomes.

## Data Availability

The data that support the findings of this study are available on request from the corresponding author [MJÖ]. The data are not publicly available due to them containing information that could compromise research participant privacy/consent.

## Abbreviations

ASA: Acetylsalicylic acid
BB: Betablocker
BP: Blood pressure
BMQ-S: Beliefs about medicines-questionnaire specific
CABG: Coronary artery by-pass graft
CL: Cholesterol lowering
CHD: Coronary heart disease
CI: Confidence interval
CVD: Cardiovascular disease
DES: Drug-eluting stent
EHR: Electronic health record
HeartQoL: Heart quality of life-instrument
ITT: Intention-to-treat
LDL-C: Low density lipoprotein-cholesterol
MAR: Missing at random
MCAR: Missing completely at random
MNAR: Missing not at random
MIMeRiC: Motivational Interviewing and Medication Review in Coronary heart disease (trial)
MMAS-8: 8-Item Morisky medication adherence scale
PCI: Percutaneous coronary intervention
PDC: Proportion of days covered
PP: Per protocol
RAAS: Renin–angiotensin–aldosterone system
RCT: Randomized controlled trial
SBP: Systolic blood pressure
SEPHIA: Secondary prevention after Heart Intensive Care Admission (quality registry)
