# Supplementary material for: Effects of a pharmaceutical care intervention on clinical outcomes and patient adherence in coronary heart disease: the MIMeRiC randomized controlled trial
Source: BMC Cardiovasc Disord. 2021 Aug 1;21:367. doi: 10.1186/s12872-021-02178-0 (PMC8327441; doi:10.1186/s12872-021-02178-0)
Supplement: Supplementary file 1 — Additional file 1. Methods Details about randomization and blinding, intervention pharmacists' education, adherence outcomes, and missing data. [file 12872_2021_2178_MOESM1_ESM.pdf]

Additional file 1 Methods

1. Randomization and blinding
2. Intervention pharmacists' education
3. Adherence outcomes and analysis
   3.1 Outcomes definition and decision tree
   3.2 Minor deviations from study protocol
4. Missing data
   4.1 Actions taken to limit the amount of missing data
   4.2 The extent of missing data
   4.3 Treatment of missing data

1. Randomization and control for bias

The randomization sequence for each stratum was computer-generated by a statistician who was not involved in data collection. An intervention to control allocation ratio of 1:1.14 was chosen to account for an expected greater loss to follow-up in the control arm. For each patient, a folded sheet of paper with the group allocation and unique study identification number written on it was kept in a sealed opaque envelope marked with a serial number; it was impossible to read the information without opening the envelope. A study administrator collected baseline questionnaires and defined the stratum of each patient before assigning the patient to the intervention or control groups according to the serial number.

Because randomization took place after the standard care process, the doctors and nurses involved in these standard visits did not know whether their patients would be in the control or the intervention group. This introduced a control for bias during the standard care period. However, this control was lost for doctors with whom the pharmacist discussed treatment during subsequent periods of the study, as it was obvious that they were discussing intervention patients, and for doctors and nurses involved in the care of those intervention patients who had further contact with the clinic after the standard follow-up. Pharmacists were not involved in any care of patients at the cardiology clinic outside of this study.

2. Intervention pharmacists' education

The intervention was performed by two clinical pharmacists (LH and MJÖ) with training in both medication review and MI. One of the pharmacists had formal specialist training in clinical pharmacy, focusing on cardiovascular medicine (60-credit Master's program in clinical pharmacy at Uppsala University, Sweden) and had completed a 15-credit course in MI from Linnaeus University, Sweden. The other had completed a 12-credit course in clinical pharmacy and pharmacotherapy from Lund University, Sweden, 2 days of internal training in MI, and a 3-day course run by a member of Motivational Interviewing Network of Trainers. Both pharmacists had carried out 5 consultations coded by Motivational Interviewing Treatment Integrity 3.1 with feedback and, in at least one of these, had been evaluated as "beginning proficiency" (≥3.5) in the global rating of MI-spirit.

3. Adherence outcomes and analysis

3.1 Outcomes definitions and decision tree

The combination of self-report (implementation) and prescription refill (persistence) of cholesterol-lowering drugs was the key secondary outcome. The patient was considered non-adherent if either method suggested non-adherence.

Refill adherence was assessed for all cholesterol-lowering drugs. Thus, patients who were prescribed both a statin and ezetimibe had to be adherent to both drug regimens to be defined as adherent.

Table 1.1. Overview of adherence measures. The primary adherence measure is shown in grey.

Drug	Method	Time	Variable	Type of adherence	Comment
**Cholesterol-lowering**	MMAS-8[a]	15 months	Discrete	Implementation	High, medium and low for cross-tabulation with refill adherence and LDL-C values
			0/1 (x)	Implementation	Cut-off ≥6 points = adherent [1,2]
	Refill	15 months	0/1 (y)	Persistence	Data collected for months 12-16, this included a time marginal; the measure related to the time point at 15 months.*
	Combined	15 months	Combined = x*y	Persistent and with high implementation	If x was missing, combined measure was 0 if y = 0.
	PDC	0-15 months	Continuous	Implementation	Not adjusted for in-hospital days
		0-15 months	0/1	Implementation	Cut-off ≥80% = adherent<br>Adjusted for in-hospital days
**Other secondary preventive drugs**	Refill	15 months	0/1	Persistence	Data collected for months 12-16, this included a time marginal; the measure related to the time point at 15 months.

Variables: 0 = no; 1 = yes

Abbreviations: MMAS-8 = 8-item Morisky Medication Adherence Scale, PDC = Proportion of days covered

* The four-month period is based on the Swedish reimbursement system [3].

[a]The use of MMAS diagnostic adherence assessment instrument is protected by US copyrighted and trademarked laws. Permission for use is required. A license is available from - MORISKY MEDICATION ADHERENCE RESEARCH, LLC., Donald E. Morisky, ScD, ScM, MSPH, MMAR, LLC, 294 Lindura Ct., Las Vegas, NV 89138; dmorisky@gmail.com.

Additional file 1 Methods

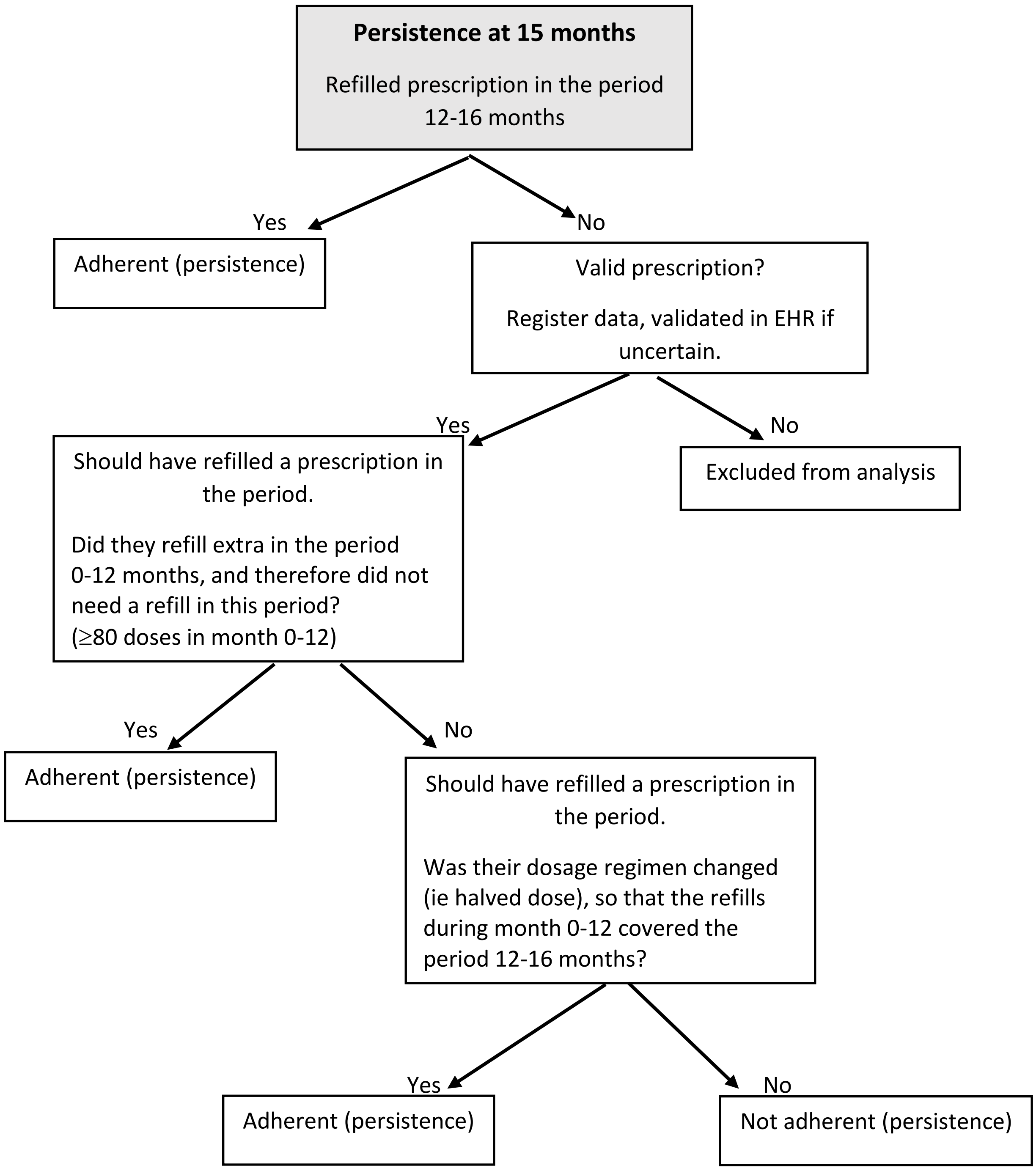

Figure 1.1. Decision tree for persistence

EHR = electronic health record;

Additional file 1 Methods

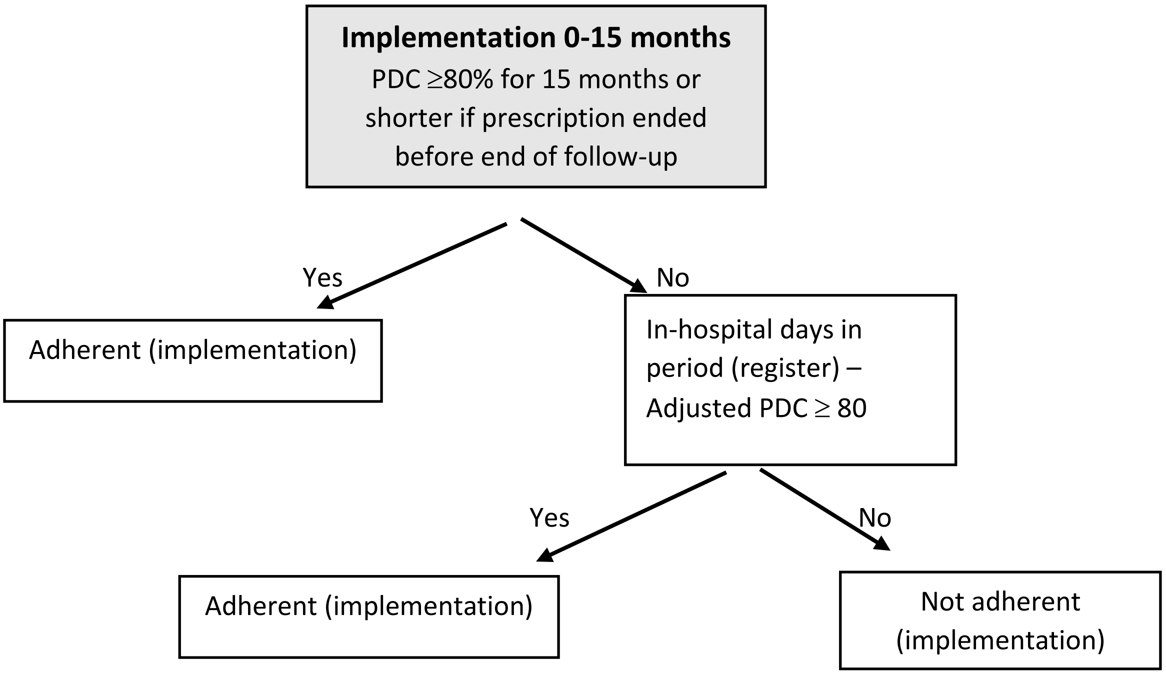

Figure 1.2. Decision tree for implementation

PDC = proportion of days covered

3.2 Minor deviations from study protocol

In the study protocol we described that we would use refill data to assess adherence to platelet aggregation inhibitor. However this was not assessed because patients were seldom prescribed these for 15 months.

As described in the study protocol, for cholesterol-lowering drug refill adherence we related the number of participants who refilled their prescriptions to the number of participants with prescriptions, which is the real patient adherence level per study group. We later choose to also relate the number of participants who refilled their prescriptions to the number of participants in the total study group. The latter relationship was used to compare the proportion of patients using drugs with that in other studies of refill adherence, where prescription data were not known.

4. Missing data

At follow up, missing data among participants was substantial. This is how we analyzed and handled the missing data.

4.1 Actions taken to limit the amount of missing data

Design:

1. We used the standard follow-up from the national quality register SWEDEHEART, which facilitated the follow-up process for the participants. The only extra effort needed from participants at follow-up was the self-reported questionnaires sent out after 15 months. We hypothesized that patients in the control group would be more reluctant to answer the questionnaire because they participated in the study but gained no treatment. We therefore sent thank-you postcards to them after receiving their questionnaire responses at baseline and 10 months. We believed that patients in the intervention group would have sufficient motivation to respond to the follow-up questionnaires because of the nature of the intervention, and so we did not send thank-you postcards to them.
2. We allowed the outcome data for blood pressure and LDL-C to be collected over a wider time period than used for the quality register, so that measurements conducted one month before and two months after the quality register interval were eligible in the trial.

Conduction of the study:

1. Patients in the intervention group who did not take part in the intervention once they had been summoned for the first or second visit were counted at first as having discontinued the trial. However, in 2017, when we realized this would imply that we had not followed the ITT analysis strategy, we contacted all these patients and asked for consent to follow-up their outcomes. From then, we treated any participants dropping out from the study in the same way.
   Two patients were not contacted, one because of a mutual understanding about withdrawal from study activities, and one because of death soon after withdrawal. 15 patients were contacted with a request for consent, and nine responded positively to this; six patients did not respond and were treated as not providing consent to further follow-up. Thus, in total 2+6 patients who were in the ITT sample did not have follow-up data. At follow-up therefore, register data could be obtained from 152 of 159 participants in the intervention group and from 156 of 157 participants oparticipants) in the control group.
2. In 2016, we realized that the administration of the quality register follow-ups had failed in our local hospital, with subsequent missing outcomes data. Thus, we decided to facilitate this administration. Missing blood pressure data dropped from 35% before this facilitation to 26% after, and missing LDL-C data dropped from 29% to 23%.

4.2 The extent of missing data

Table 1.2. Details about missing data and possible causes

**Outcome**	**Missing data %** Control/ Intervention	**Expected reasons for missing data**	**Assumption**
Blood pressure	31/30	Failed administration in the quality register or in the trial	MCAR
		Patient failed to show up for the test – accidentally because they had health issues that prevented them or they forgot, or deliberately because they did not want to be followed-up or they did not find it relevant.	MAR - MNAR
LDL-C	28/25	Failed administration in the quality register or in the trial	MCAR
		Patient failed to show up for the test – accidentally because they had health issues that prevented them or they forgot, or deliberately because they did not want to be followed-up or they did not find it relevant.	MAR - MNAR
MMAS-8 and HeartQoL	13/19	Failed administration of questionnaires (9 in control, 5 in intervention)	MCAR
	13/22	Patients forgot to respond to questionnaires;<br>had low motivation and did not respond to questionnaires; or chose not to respond to questionnaires because they were not satisfied with their treatment.<br>The difference between control and intervention patients could have been the result of the intervention patients not receiving a thank you postcard as an affirmation of their contribution. Alternatively, patients in the intervention group may have chosen not to respond because they did not like the intervention activities they had experienced or did not want to disappoint the pharmacist with a negative attitude or low adherence.	MAR - MNAR
Refill adherence and hospital contacts (register data)	0.5/4.5	Patients decided to withdraw from the study and did not respond to our letter about continued consent for follow-up in the register and EHR.<br><br>These patients might have found the intervention either not relevant to them, or needing too much effort from them. They may not have responded to our second consent question because of forgetfulness, because they did not find the study relevant, because they assumed that their outcomes would be bad (social desirability), or because they considered it a violation of their personal integrity as they had in fact withdrawn from the study.	MAR/MNAR

EHR = electronic health record; Heartqol = health-related quality of life; LDL-C = low-density lipoprotein cholesterol; MAR = missing at random; MCAR = missing completely at random; MMAS-8 = Morisky 8-item adherence scale; MNAR = missing not at random.

4.3 Treatment of missing data

As recommended if there is a univariate outcome[4] and no strong auxiliary variables[4,5], we decided to conduct a complete case analysis adjusted for covariates associated with missing data (i.e. MCAR but adjusted towards MAR). We explored the impacts of MNAR assumptions in sensitivity analyses. We conducted sensitivity analyses for the primary outcome, LDL-C, and for the secondary outcomes with significant differences between the groups, in order to control for type I errors.

Characteristics of participants with one or more missing outcomes did not differ significantly from those of participants with complete outcome data. However, some differences were found for individual outcomes, as described in the table below:

Table 1.3. Differences in baseline characteristics for patients with missing outcome data compared to patients with full outcome data

Missing outcome	Significant differences (missing data vs full data; $p < 0.05$)
Low-density lipoprotein cholesterol	Mean age (SD): 65.9 (10.8) years vs 69.4 (7.7) years<br>Living alone: 26.5% vs 19%
Blood pressure	Mean age (SD): 67.4 (10.6) years vs 68.9 (7.7) years

We adjusted the analysis of the primary outcome for baseline covariates that differed between participants with full outcome data and those with missing data, i.e. age and civil status. Since this did not affect the overall outcome, we reported the unadjusted result only and decided not to use the adjustment in any of the following analyses.

Missing data were almost exclusively related to the clinical results and questionnaires. However, eight patients (seven in the intervention group) were not found on the register at all, i.e. data were completely missing. These were the eight patients described in "Missing data – conduction of the study". Although there were very few missing patients, because most of them were in the intervention group, we cannot exclude that this may have affected the results for adherence outcomes. We therefore described the variables assumed to be most strongly connected to adherence at baseline in order to guide the interpretation of the sensitivity analyses (Table Biii). Statistical tests were not performed because of the low number of participants.

Table 1.4. Variables associated with adherence at baseline in patients missing at follow-up compared to patients still participating at follow-up

Variable at baseline	Patients missing at follow-up (n=8)	Patients still participating at follow-up (n=298)
MMAS-8 high or medium score, n (%)	8 (100)	261 (92.2)
BMQ Necessity score	18.1 (2.9)	19.0 (3.1)
BMQ Concern score	12.3 (4.0)	13.1 (5.0)
BMQ Attitudinal group	4 (50%) of patients had an accepting attitude	157 (53%) of patients had an accepting attitude

BMQ = Beliefs about Medicines Questionnaire; MMAS-8 = Morisky 8-item adherence scale.

References

1. Morisky DE, Ang A, Krousel-Wood M, Ward HJ. Predictive validity of a medication adherence measure in an outpatient setting. *J Clin Hypertens (Greenwich).* 2008;10(5):348-354.
2. Krousel-Wood M, Holt E, Joyce C, et al. Differences in cardiovascular disease risk when antihypertensive medication adherence is assessed by pharmacy fill versus self-report: the Cohort Study of Medication Adherence among Older Adults (CoSMO). *Journal of hypertension.* 2015;33(2):412-420.
3. Glader EL, Sjolander M, Eriksson M, Lundberg M. Persistent use of secondary preventive drugs declines rapidly during the first 2 years after stroke. *Stroke.* 2010;41(2):397-401.
4. Sullivan TR, White IR, Salter AB, Ryan P, Lee KJ. Should multiple imputation be the method of choice for handling missing data in randomized trials? *Stat Methods Med Res.* 2018;27(9):2610-2626.
5. White IR, Carpenter J, Horton NJ. Including all individuals is not enough: lessons for intention-to-treat analysis. *Clin Trials.* 2012;9(4):396-407.
