# Supplementary material for: Effects of a pharmaceutical care intervention on clinical outcomes and patient adherence in coronary heart disease: the MIMeRiC randomized controlled trial
Source: BMC Cardiovasc Disord. 2021 Aug 1;21:367. doi: 10.1186/s12872-021-02178-0 (PMC8327441; doi:10.1186/s12872-021-02178-0)
Supplement: Supplementary file 2 — Additional file 2. Additional results Characteristics of non-participants, drugs prescribed at discharge, participants and activities in intervention, relation between adherence measures and between adherence and LDL-C, per-protocol analysis. [file 12872_2021_2178_MOESM2_ESM.pdf]

Additional file 2. Additional results

1. Characteristics of non-participants
2. Drugs prescribed at discharge
3. Participants and activities in intervention
4. Relation between adherence measures and between adherence and LDL-C
5. Per protocol analysis

1. Characteristics of non-participants

Non-participants were those who could not be reached, or declined to participate. They were of similar age, were as likely to have had earlier CHD, and were as likely to have acute CHD at the time of enrollment as those who participated. However, non-participants were more likely to live alone (48% vs 21%; p<0.01). The reasons for not participating included, not wanting the long travel (those living far from the hospital), not wanting the extra visits, feeling too old, having had the medicines before, and/or feeling satisfied with standard care.

Patients who consented to participate but then failed to return their baseline questionnaires (n=69) and therefore were not randomized differed from those who did fully participate in that they were younger (65.2±13.7 vs 68.5±8.7 years; p<0.05) and were less likely to have had higher education (17.5% vs 38.0%; p<0.01), and they also had higher LDL-C levels at baseline (3.43±1.24 vs 2.25±0.77 mmol/L; p<0.01), see Table 2.1.

Table 2.1. Baseline characteristics of participants and non-participants

	**Participants (n=316)**	**Consented but did not return baseline questionnaires (n=68)**	**Non-participants (n=248)**
Age (y), mean ± SD	68.5 (8.9)	65.2 (13.6)*	68.9 (10.1)
Male, n (%)	235 (74.4)	52 (77.6)	186 (75.0)
Born outside Sweden, n (%)	10 (6.3)	4 (5.9)	
Married or cohabitating, n (%) †	126 (79.2)	51 (75.0)	102 (51.5) ‡
Educational level, n (%)			
Comprehensive school	56 (35.2)	28 (41.2)	
Upper secondary school	43 (27.0)	28 (41.2)	
Bachelor's/Master's degree	120 (38.0)	12 (17.6) ‡	
Acute coronary syndrome, n (%)	220 (69.6)	48 (70.5)	
History of CHD, n (%)	92 (29.1)	21 (30.1)	
LDL-C at baseline, mmol/L	2.25 (0.77)	3.43 (1.24) ‡	

* P < .05 vs participants,

† Note that this factor was known only among less than 50% of those who did not consent,

‡ P < .01 vs participants

2. Drugs prescribed at discharge

Table 2.2. Drugs prescribed at discharge

	Intervention group, n (%)	Control group, n (%)	*P*
ASA (Aspirin)	135 (85.4)	140 (90.3)	.186
P2Y12-inhibitor	146 (92.4)	131 (84.5)	.047
Clopidogrel	53 (33.5)	55 (35.5)	
Ticagrelor	92 (58.2)	76 (49.0)	
Warfarin or NOAC	23 (14.6)	15 (9.7)	.140
ACE-inhibitor	81 (51.39	80 (51.6)	.951
Angiotensin-II blocker	65 (41.1)	61 (39.4)	.748
Betablocking agents	145 (91.8)	141 (91.0)	.800
Statin	151 (95.6)	153 (98.7)	.097
Atorvastatin	122 (76.7)	125 (80.1)	
Pravastatin	2 (1.3)	3 (1.9)	
Simvastatin	23 (14.5)	23 (14.7)	
Rosuvastatin	4 (2.5)	3 (1.9)	
High-intensity statin*	117 (74.1)	113 (72.9)	.818
Ezetimibe	4 (2.5)	3 (1.9)	.721
Diuretics	17 (10.8)	22 (14.3)	.357
Other antihypertensives	43 (27.2)	38 (24.5)	.586
Long-acting nitrates	15 (9.5)	5 (3.2)	.023
Insulin	15 (9.5)	13 (8.4)	.732
Oral antidiabetics	22 (13.9)	21 (13.5)	.682

Abbreviations: ACE, angiotensin-converting enzyme; ARB, angiotensin receptor blockers; ASA, acetylsalicylic acid; BB, beta-blocking agent; n, number; NOAC, new oral anticoagulant.
*High intensity statin defined as atorvastatin dosage ≥ 40mg/day or rosuvastatin dosage ≥ 20mg/day

3. Participants and activities in the intervention

All patients randomized to intervention (n=159) were summoned for a first visit, and 144 completed this, a flowchart of intervention activities is outlined in figure Bi. After the follow-up at 14 days, patients received either the basic intervention (n=55) or, based on need, the intensive intervention (n=82). Most extra contacts in the intensive intervention were conducted by phone, and most patients with the intensive intervention (n=59) had one or two extra follow-up contacts. The majority of all the intervention patients (n=123) also completed the final pre-planned follow-up at 10 months.

Additional file 2. Additional results

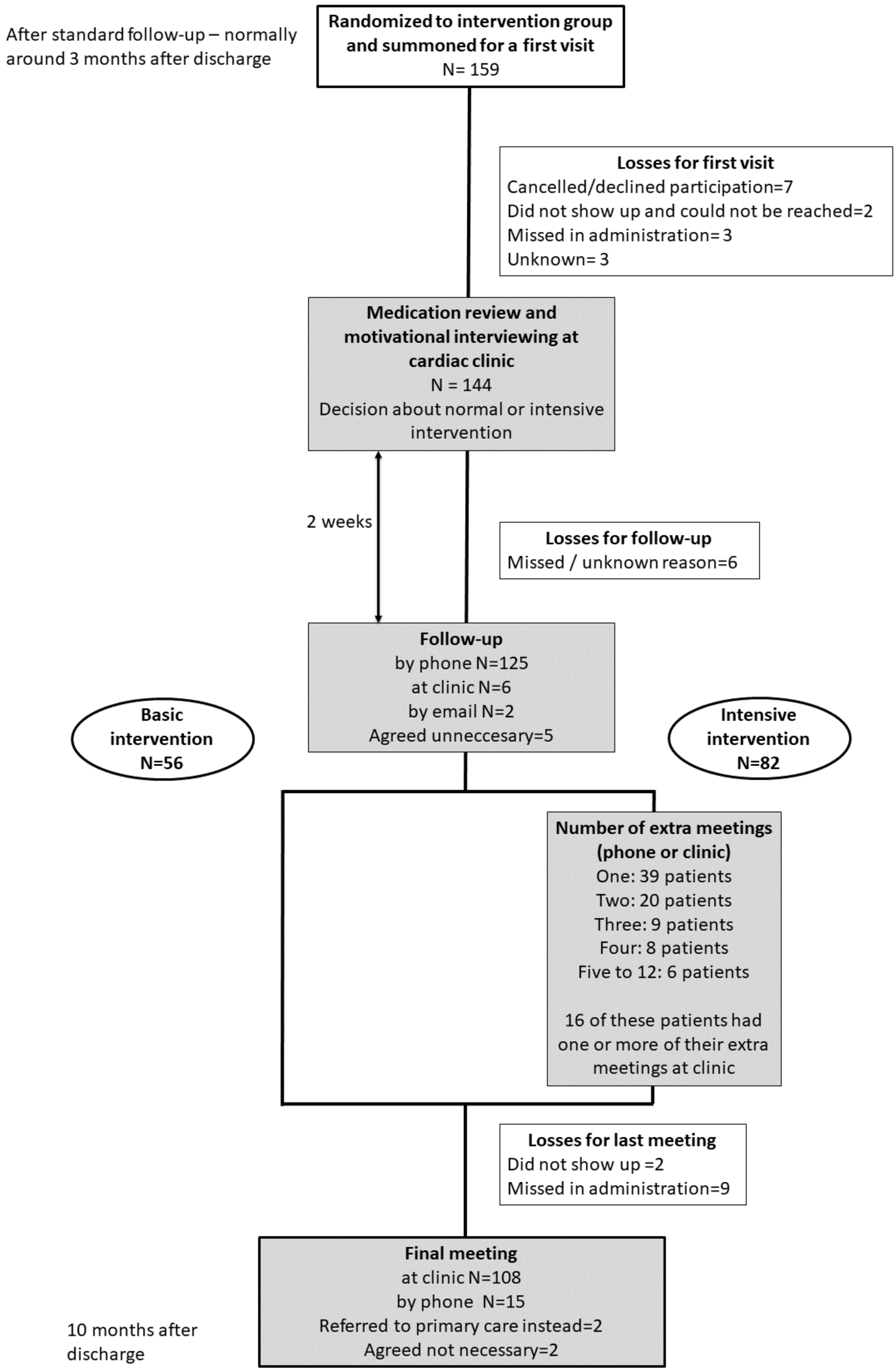

Figure 2.1 Flow chart of intervention

4. Relation between adherence measures and between adherence and LDL-C

Table 2.3. Cross tabulation of adherence measures at 15 months

Refill adherent	Participants with MMAS-8[a] score (n)	Low MMAS-8 score	Medium MMAS-8 score	High MMAS-8 score
**No**	22	5 (22.7%)	3 (13.6%)	14 (63.6%)
**Yes**	219	15 (6.8%)	59 (26.9%)	145 (66.2%)
		*P** = .025		

MMAS-8, Morisky 8-item adherence scale; n, number;
*Fishers exact test for low vs medium/high MMAS-8 score.

[a]The use of MMAS diagnostic adherence assessment instrument is protected by US copyrighted and trademarked laws. Permission for use is required. A license is available from - MORISKY MEDICATION ADHERENCE RESEARCH, LLC., Donald E. Morisky, ScD, ScM, MSPH, MMAR, LLC, 294 Lindura Ct., Las Vegas, NV 89138; dmorisky@gmail.com.

Table 2.4. Relationship between dichotomous PDC and LDL-C outcomes in the intervention and control groups

	Intervention group			Control group		
	Not adherent to CL-drug	Adherent to CL-drug	*P*	Not adherent to CL-drug	Adherent to CL-drug	*P*
Participants, n (%)	20 (18.0)	91 (82.0)		25 (20.7)	86 (79.3)	
LDL-C <1.8 mmol/L, %	25.0	41.8	.164*	9 36.0	47.7	.302*
LDL-C (mmol/L), mean (SD)	2.5 (0.7)	2.1 (0.7)	.049†	2.2 (0.7)	2.3 (0.7)	.495†

Variables: Not adherent = PDC<80%; adherent = PDC ≥80%
CL = cholesterol-lowering; LDL-C = low-density lipoprotein cholesterol. *Chi2-test; †Independent sample *t* test.

5. Per-protocol analysis

The results of the adherence tests were reinforced for the intervention group when only patients who had received the full intervention were included, i.e per protocol (n=131). According to the combined measure, adherence was achieved for 89.2% of the intervention patients, resulting in an absolute risk difference of 11.8 % (95% CI 2.5% to 21.1%; *P* = .019) vs the control group. For those prescribed aspirin, 97.4% of the intervention group patients were persistent, resulting in an absolute risk difference of 6.2 % (95% CI 0.8% to 11.7%; *P* = .038) vs the control group). For those prescribed beta-blocking agents or RAAS inhibitors, the proportion of patients who were persistent also increased in the intervention group versus the ITT analysis, but there were no evident differences between the intervention and control groups. For secondary care use, the proportion of patients who had unplanned contacts was increased in the intervention group versus the ITT analysis and the resulting absolute difference between intervention and control group was 7.4% (95% CI -0.5% to 15.2%; *P* = .061). There were no other evident changes in clinical outcomes.
