# Supplementary material for: Effects of a pharmaceutical care intervention on clinical outcomes and patient adherence in coronary heart disease: the MIMeRiC randomized controlled trial
Source: BMC Cardiovasc Disord. 2021 Aug 1;21:367. doi: 10.1186/s12872-021-02178-0 (PMC8327441; doi:10.1186/s12872-021-02178-0)
Supplement: Supplementary file 3 — Additional file 3. Sensitivity analyses of primary outcome and relevant secondary outcomes. [file 12872_2021_2178_MOESM3_ESM.pdf]

Sensitivity analyses

Sensitivity analyses carried out to explore the impact of missing data are presented in table Ci-iii below. We also performed sensitivity analyses to explore the robustness of the analyses in relation to outcomes definitions for achievement of the LDL-C goal (table 3.1), and secondary healthcare use (table 3.4). The latter was carried out because of a significant difference in this measure in the per-protocol analysis, and because the ICD code definition for suspected myocardial infarction is on the margin of the definition for coronary heart disease and also seemed to differ between the groups.

When missing LDL-C values in each group were imputed from the rates of goal achievement in participants with observed values in the respective groups (37.0% in the intervention group and 44.2% in controls), i.e. increasing the sample size, the absolute risk difference was -7.3% (95% CI -18.1% to 3.5%) and when the LDL-C concentrations in patients with missing values were imputed with goal achievement <30% in intervention and >50% in control patients, the control group had a better outcome (Table Ci). On the other hand, goal achievement rates would have had to differ substantially among those with missing values in the two groups to alter the conclusion to a positive result for the intervention group. Sensitivity analysis of the cut-off point for reaching LDL-C goal achievement did not change the result of the primary outcome.

Sensitivity analyses were also carried out for the two adherence measures with significant risk differences, and the results were found to be robust to different assumptions about missing data (Table 3.2-3.3). Sensitivity analysis of secondary healthcare use was also carried out because the number of patients diagnosed as ICD-10 code Z03.4 (“observation for suspected myocardial infarction”) differed substantially between the groups. When Z03.4 was excluded, the absolute difference between intervention and control group patients was reduced in both the ITT (1.5%, 95% CI -4.8% to 7.7%) and per-protocol (3.2%, 95% CI -3.7% to 10.0%) analyses (Table 3.4).

Table 3.1. Sensitivity analyses LDL-C goal achievement (<1.8 mmol/L)

Tests	Intervention n=159	Control n=157	Risk difference %	Risk difference 95% CI	*P*	Assumptions
Main analysis (for reference)	*No imputation*		*-7.2*	*-19.9 to 7.3*	*.263*	*Participants with missing values had the same rate of goal achievement as participants with no missing values (MCAR).*
Number of participants with missing data (n)	*40*	*44*				
Goal achievement rate imputed (%)	40.5	40.5	-5.3	-16.1 to 5.5	.332	Same rate in participants with missing and no missing values, no real difference between control and intervention groups (mean of both groups used for imputation of missing values).
Goal achievement rate imputed (%)	37	44.2	-7.3	-18.1 to 3.5	.190	Same rate in participants with missing and no missing values, using the same difference between groups as in participants with no missing values (mean of each group used for imputation of missing values). That is, only increasing sample size.

Goal achievement rate imputed (%)[a]	30	51	-10.9	-21.7 to 0.1	.049	Intervention: The rate of goal achievement (30%) in participants with missing values was slightly lower than in those with no missing values. Control: The reverse was assumed (51% goal achievement in participants with missing values).
Goal achievement rate imputed (%)[b]	78.0	15	15.0	4.2 to -25.8	.043	Intervention: The rate of goal achievement (78%) in participants with missing values was substantially higher than in those with no missing values. Control: The reverse was assumed (15% goal achievement in participants with missing values).
LDL-C goal ≤1.8[c]	No imputation		-7.5	-20.3 to 5.3	.254	≤1.8 mmol/L was an acceptable goal achievement.
LDL-C goal ≤2.0	No imputation		-8.7	-21.5 to 4.1	.183	≤2.0 mmol/L was an acceptable goal achievement.

Abbreviations: LDL-C = low-density lipoprotein cholesterol; MCAR = data missing completely at random

a Test of what would have been needed to make the difference significant in favor of the control group.

b Test of what would have been needed to make the difference significant in favor of the intervention group.

c Test of the sensitivity of the cut-off point for goal achievement.

Table 3.2. Sensitivity analysis of refill adherence

Tests	Intervention n=159	Control n=157	Risk difference		*P*	Assumptions
			%	95% CI		
Main analysis (for reference)	*No imputation*		*8.5*	*1.7 to 15.3*	*.017*	*Participants with missing values had the same adherence rates as participants with no missing values (MCAR).*
Number of participants with missing data	*7*	*1*				
Number of participants with missing data imputed as nonadherent	1	0	7.4	0.7 to 14.1	.033	Intervention: Rate of refill adherence 85.7% in participants with missing values and 94.3% in those with no missing values. Control: Full adherence in the individual with missing values. Overall population: Rate 90%
Number of participants with missing data imputed as nonadherent	2	0	6.7	-0.1 to 13.6	.057	Intervention: Rate of refill adherence 71.4% in participants with missing values and 94.3% in those with no missing values. Control: Full adherence in the individual with missing values. Overall population: Rate 90%

Abbreviations: MCAR = data missing completely at random

Table 3.2. Sensitivity analysis of combined measure of adherence

Tests	Intervention n=159	Control n=157	Risk difference %	Risk difference 95% CI	*P*	Assumptions
Main analysis (for reference)	*No imputation*		*10.4*	*1.1 to 19.7*	*.033*	*Participants with missing values were as adherent as participants with no missing values (MCAR).*
Number of participants with missing data	*31*	*16*				
Number of participants with missing data imputed as nonadherent	3	2	9.9	1.5 to 18.3	.022	Intervention and Control: Same rate of self-reported non-adherence (≈10%). Same rate in participants with missing MMAS data as in participants with no missing MMAS data.
Number of participants with missing data imputed as nonadherent	4	2	9.2	0.8 to 17.7	.035	Intervention: Higher rate of self-reported non-adherence (13.0%) in participants with missing MMAS data than in those with no missing data. Control: Same rate of self-reported non-adherence in participants with and without missing MMAS data.
Number of participants with missing data imputed as nonadherent	5	2	8.6	0.0 to 17.1	.051	Intervention: Higher rate of self-reported non-adherence (16.0%) in participants with missing MMAS data than in those with no missing data. Control: Same rate of self-reported non-adherence in participants with and without missing MMAS data.
Number of participants with missing data imputed as nonadherent	6	3	8.5	-0.1 to 17.2	.056	Intervention and Control: Same rate of self-reported non-adherence (≈19%); rate in participants with missing MMAS data was twice that in participants with no missing MMAS data.

Abbreviations: MCAR = data missing completely at random; MMAS = Morisky 8-item adherence scale

Table 3.4. Sensitivity analyses secondary healthcare use

Tests	Intervention n=159	Control n=157	Risk difference %	Risk difference 95% CI	*P*	Assumptions
Main analysis ITT (for reference)	*No imputation*		*5.4*	*-1.7 to 12.6*	*.138*	*Contacts diagnosed as I00-I99 and Z034,*
Main analysis PP (for reference)	*No imputation*		*7.4*	*-0.5 to 15.2*	*.061*	
ITT excluding Z03.4	No imputation		1.5	-4.8 to 7.7	.646	Z03.4 is not a good measure of CHD
PP excluding Z03.4	No imputation		3.2	-3.7 to 10.0	.358	

Abbreviations: ITT = intention-to-treat statistical analysis; PP = per-protocol statistical analysis; Z03.4 = ICD10 code ’observation for suspected myocardial infarction’.
